# POSTTRAUMATIC GROWTH DURING COVID PANDEMIC AMONG OLDER ADULTS: A MIXED METHODS STUDY

**DOI:** 10.1093/geroni/igad104.2054

**Published:** 2023-12-21

**Authors:** Lenny Chiang-Hanisko, Patricia Liehr

**Affiliations:** Florida Atlantic University, Boca Raton, Florida, United States; Florida Atlantic University, Boca Raton, Florida, United States

## Abstract

COVID has impacted life for the older adult population living in residential settings affecting psycho-social well-being. Risk for COVID is associated with increasing age and pre-existing health conditions. The purpose of the study was to investigate the post-traumatic growth (PTG) experience among older adults, and the impact of COVID and associated regulatory precautions, on well-being for older adults living in a continue care community (CCC). A parallel mixed-methods approach was used to explore the experience of older adults with PTG. Quantitative data were analyzed using descriptive statistics. Qualitative data were analyzed using content analysis to identify emergent themes most frequently reported from open-ended responses. Older adults (N=98) living in a single CCC completed the survey. Age ranged from 67-99 years (mean = 86) with over 90% identifying as non-Hispanic White; and female (75%). Over half (51%) reported social-psychological issues, including anxiety (32%) and depression (31%); 41% reported knowing residents who died during COVID, attributed to will to live (35%), social isolation (30%) and coexisting health conditions (75%). Qualitative analysis revealed 13 subthemes related to 5 major PTG concepts: New possibilities, relating to others, personal strength, spiritual growth and appreciation for life. This study provides a deeper understanding of how older adults residing in a CCC coped with COVID and their experiences. Results indicated that older adults experienced positive changes and PTG despite challenges. Healthcare providers should recognize these potential positive changes and growth and use them as a source for improving older adult well-being when facing pandemic challenges.

